# MRI Feature Tracking Strain in Pulmonary Hypertension: Utility of Combined Left Atrial Volumetric and Deformation Assessment in Distinguishing Post- From Pre-capillary Physiology

**DOI:** 10.3389/fcvm.2022.787656

**Published:** 2022-03-17

**Authors:** Kai'En Leong, Luke Howard, Francesco Lo Giudice, Holly Pavey, Rachel Davies, Gulammehdi Haji, Simon Gibbs, Deepa Gopalan

**Affiliations:** ^1^Department of Radiology, Imperial College National Health Service Trust/Hammersmith Hospital, London, United Kingdom; ^2^Department of Cardiology, The Royal Melbourne Hospital, Parkville, VIC, Australia; ^3^National Pulmonary Hypertension Service, Imperial College National Health Service Trust, London, United Kingdom; ^4^National Heart and Lung Institute, Imperial College London, London, United Kingdom; ^5^Department of Cardiology, Imperial College National Health Service Trust/Hammersmith Hospital, London, United Kingdom; ^6^Division of Experimental Medicine and Immunotherapeutics, University of Cambridge, Cambridge, United Kingdom; ^7^Department of Radiology, Cambridge University Hospitals National Health Service Trust, Cambridge, United Kingdom

**Keywords:** left atrial strain (LA strain), pulmonary hypertension, pre-capillary pulmonary hypertension, post-capillary pulmonary hypertension, feature tracking (CMR-FT), cardiac MRI (CMR)

## Abstract

**Aims:**

Pulmonary hypertension (PH) is dichotomized into pre- and post-capillary physiology by invasive catheterization. Imaging, particularly strain assessment, may aid in classification and be helpful with ambiguous hemodynamics. We sought to define cardiac MRI (CMR) feature tracking biatrial peak reservoir and biventricular peak systolic strain in pre- and post-capillary PH and examine the performance of peak left atrial strain in distinguishing the 2 groups compared to TTE.

**Methods and Results:**

Retrospective cross-sectional study from 1 Jan 2015 to 31 Dec 2020; 48 patients (22 pre- and 26 post-capillary) were included with contemporaneous TTE, CMR and catheterization. Mean pulmonary artery pressures were higher in the pre-capillary cohort (55 ± 14 vs. 42 ± 9 mmHg; *p* < 0.001) as was pulmonary vascular resistance (median 11.7 vs. 3.7 WU; *p* < 0.001). Post-capillary patients had significantly larger left atria (60 ± 22 vs. 25 ± 9 ml/m^2^; *p* < 0.001). There was no difference in right atrial volumes between groups (60 ± 21 vs. 61 ± 29 ml/m^2^; *p* = 0.694), however peak RA strain was lower in post-capillary PH patients (8.9 ± 5.5 vs. 18.8 ± 7.0%; *p* < 0.001). In the post-capillary group, there was commensurately severe peak strain impairment in both atria (LA strain 9.0 ± 5.8%, RA strain 8.9 ± 5.5%). CMR LAVi and peak LA strain had a multivariate AUC of 0.98 (95% CI 0.89–1.00; *p* < 0.001) for post-capillary PH diagnosis which was superior to TTE.

**Conclusion:**

CMR volumetric and deformation assessment of the left atrium can highly accurately distinguish post- from pre-capillary PH.

## Introduction

Pulmonary hypertension (PH) is a hemodynamic diagnosis with diverse etiologies and variable prognoses (1). Dichotomisation into pre- and post-capillary physiology is based on left ventricular (LV) filling pressures (mean pulmonary arterial wedge pressure/mPAWP). Consequently, right heart catheterization remains the reference standard (1).

Pre-capillary (mPAWP ≤ 15 mmHg) PH encompasses most of the WHO classification groups (1, 3, 4, and 5) while post-capillary PH (mPAWP > 15 mmHg) relates to left heart disease (group 2) (1–3).

Guidelines (1) have suggested clinical, echocardiographic (TTE) and ECG features that increase pre-catheterization suspicion for post-capillary PH. However, these are inadequately sensitive/specific, prompting development of additional imaging parameters to enhance discrimination. With TTE, Scalia and colleagues (4) proposed ePLAR (ratio of TR Vmax to E/medial e') and Venkateshvaran et al. (5) described ePLAGS (ratio of TR Vmax to peak LA strain) with high predictive ability. A single parameter of cardiac MRI (CMR) assessed LAVi < 43 ml/m^2^ has been shown to have excellent performance in distinguishing IPAH from HFpEF (6). With non-gated CT, reduced LA size with a trans-axial LA/RA ratio < 0.7 identified pre-capillary PH from HFpEF patients (7).

Contemporarily, interest in deformation imaging has increased with strain assessment offering additive diagnostic and prognostic value in various pathologies (8, 9). However, most data relate to the echocardiographic speckle tracking technique, although CMR feature tracking (FT) strain appears reasonably concordant (10). Reduced peak LA reservoir strain has been shown to have superior correlation with elevated LV filling pressures over standard parameters like E/e' and LAVi (11–13). Accordingly, with pre- and post-capillary dichotomization contingent on invasive mPAWP measurement, it is plausible that LA strain may be the optimal imaging surrogate.

Importantly, left and right atrial deformation has mainly been examined separately and the pattern of biatrial impairment in pre- vs. post-capillary PH is poorly defined.

In this context, we sought to first characterize CMR FT biatrial peak reservoir and biventricular peak systolic strain in pre- and post-capillary PH patients and then examine the performance of peak LA strain in distinguishing the 2 groups compared to other discriminatory parameters (4, 6).

## Methods

### Patients

This was a retrospective cross-sectional study with all patients referred to a single high volume PH center for PH assessment between 1 January 2015 and 31 December 2020 screened for eligibility. Only consecutive patients who had all investigations (CMR, TTE and catheterization) performed by our service were included. Patients were excluded if any of the 3 tests were performed prior to referral and not repeated by us. This was to minimize random error and bias arising from potential methodological differences at external sites. Medical records were reviewed to determine PH classification groups according to current guidelines (1), atrial fibrillation (AF) and medication history and functional class.

This study has been approved by the Heath Research Authority (HRA) and NHS Research Ethics Service (REC) (IRAS ID 280472/Protocol 20HH5838) with waivers of informed consent due to the retrospective design.

### Right Heart Catheterization

Patients were allowed only oral clear fluids prior to catheterization. This was to avoid dehydration and factitious PAWP normalization. Vascular access was via right internal jugular (ultrasound-guided) or occasionally right antecubital venous puncture. A Swan-Ganz catheter was used for all pressure measurements (mean of 3 readings recorded in sinus rhythm or 5 in AF). PAWP tracings were obtained in end-expiration. Cardiac output (CO) measurements were made using the thermodilution or Fick method. PVR (Wood units/WU) was calculated by (mPAP-mPAWP) ÷ CO.

### Hemodynamic Definitions

Patients were classified as having pre-capillary PH if their invasive mPAP was ≥ 25 mmHg and mPAWP ≤ 15 mmHg. A designation of post-capillary PH was applied if the mPAWP was >15 mmHg (1).

### TTE and ePLAR

All patients had Doppler parameters and left atrial volume (LAV) measured in accordance with current guidelines (14). ePLAR was selected as the echocardiographic comparator due to its integration of multiple Doppler estimates of hemodynamic parameters and was calculated as described: ePLAR = TR Vmax ÷ E/medial e' (4).

Reduced medial (but preserved lateral) mitral annular e' velocities may be observed in pre-capillary PH (15) and can cause incorrect conclusion of raised LV filling pressures when the E/e' parameter is computed using the medial e' velocity. As such, guidelines propose utilization of E/lateral e' for estimation of LV filling pressures in pre-capillary PH (16). Accordingly, we calculated additional “ePLAR variants” using the lateral e' velocity (TR Vmax ÷ E/lateral e') and the average (medial and lateral) E/e' (TR Vmax ÷ average E/e').

### CMR

All patients underwent comprehensive CMR on a 1.5T Siemens Aera scanner (Erlangen, Germany) using a 32-channel phased array surface coil as part of routine care. Cine imaging was performed with breath-held steady state free precession sequences to derive contiguous parallel short axis slices of both ventricles from base to apex, as well as standard long axis slices of the heart. Slice prescription parameters were 8 mm thickness/2 mm gap for short axis imaging with 1.5–2 mm in-plane spatial resolution, and 33–45 ms temporal resolution with 25–30 reconstructed cardiac phases.

### CMR Post Processing and FT Strain

CMR studies were analyzed using Circle Cardiovascular Imaging (CVI42, version 5.12.1, Calgary, Canada). Volumetric analysis was performed as per guidelines (17).

**Figure 1:**
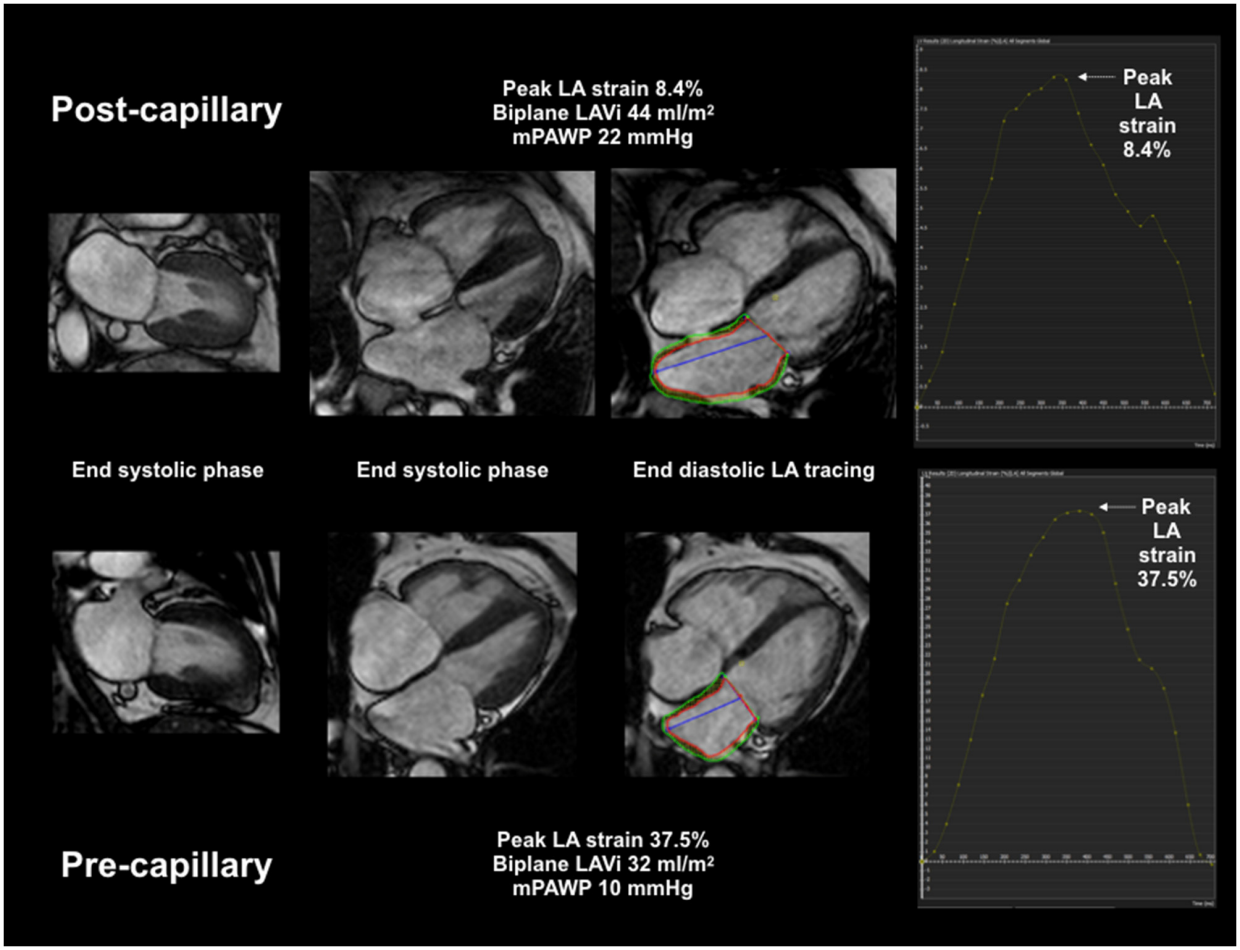
CMR LA volumetric and deformation assessment. Combined CMR LAVi and peak LA strain multivariate AUC 0.98 for post-capillary PH diagnosis.

Myocardial and atrial deformation FT was also performed using CVI42 (Figure 1). Endocardial contours were traced using automatic detection by “3 click” (2 at base, 1 at apex) definition of chamber extent. Minor manual endocardial adjustment was applied as required. Epicardial boundaries were traced manually. Chamber borders were traced at ventricular end-diastole to define strain zero baseline (R-R gating) and longitudinal strain quantification was performed automatically by the software using a deformable myocardial model (10, 18–20). LA reservoir strain and LV global longitudinal strain (GLS) were computed using the 4 and 2 chamber cines while only the 4 chamber cine was used for RA strain and RV strain.

With regard to the RV, only the free wall endo- and epicardial borders were traced to derive the RV free wall longitudinal strain (RVFWLS), with tracing and deformation of the septum assigned to the LV.

Three repeat measurements of peak biatrial reservoir, LV GLS and RVFWLS were performed, and the average recorded.

Ten patients were randomly selected for strain measurement by a second investigator (DG) to assess inter-observer variability. Intra-observer variability was assessed with one investigator (KL) re-measuring atrial and ventricular strain after 1 month.

### Statistical Analysis

Continuous normal data are presented as mean (±1 standard deviation). Non-normal data are presented as median (inter-quartile range). Continuous data was compared with the two tailed Student's *t*-test or Mann Whitney U test for normal and non-normal data, respectively. Categorical variables are displayed as n (%) and differences assessed using Fisher's exact test. Intraclass correlation coefficient (ICC) estimates and their 95% confidence intervals for inter- and intra-observer variability in strain assessment were calculated. Correlation analysis was performed using Pearson correlation coefficient or Spearman rank test where data was non-normal. TTE and CMR measurement of LAV was compared with Bland-Altman analysis. Univariate and multivariate stepwise logistic regression was performed to identify variables that differentiated post- from pre-capillary PH. Receiver operating characteristic (ROC) curves with area under the curve (AUC) values were used to compare discriminative ability of the various parameters and the optimal cut-points were defined by the Youden index. A *p*-value of ≤0.05 was considered statistically significant.

## Results

### CMR FT Strain Reproducibility

Measurement of FT biatrial and biventricular strain was feasible in all patients. There was excellent inter- and intra-observer agreement. Intra-observer (KL) ICC for the RA, LA, RV and LV were 0.99 (0.97–1.0), 1.0 (0.99–1.0), 0.99 (0.95–1.0) and 0.97 (0.87–0.99), respectively.

Inter-observer (DG, KL) ICC were 0.98 for RA (0.90–0.99), 1.0 for LA (0.98–1.0), 0.99 for RV (0.96–1.0) and 0.97 for LV (0.88–0.99).

### Patient Characteristics

A total of 48 patients were included (22 pre- and 26 post-capillary). Pre-capillary PH patients were mainly female and younger, with lower BMI and preserved renal function. Only 1 pre-capillary PH patient had an AF history compared to 20 (77%) post-capillary patients (*p* < 0.001) (Table 1).

**Table 1:** Baseline patient characteristics.

	**Pre-capillary** **(*n* = 22)**	**Post-capillary** **(*n* = 26)**	***p*-value**
Age (years)	48 ± 17	71 ± 11	**<0.001**
Sex (%)	Male 6 (27%)	Male 12 (46%)	0.237
	Female 16 (73%)	Female 14 (54%)	
BMI (kg/m^2^)	27.5 ± 7.4	33.1 ± 7.4	**0.009**
Serum creatinine (μmol/L)	77 ± 15	114 ± 74	**0.007**
AF at TTE (%)	0	15 (58%)	**<0.001**
Any history of documented AF (%)	1 (5%)	20 (77%)	**<0.001**
Clinical classification	Idiopathic PAH – 14 (64%) Heritable PAH – 1 (4.5%) CTD associated PAH – 4 (18%) HIV associated PAH – 1 (4.5%) CHD associated PAH – 2 (9%)	SD – 1 (4%) DD – 25 (96%)	**–**
Functional class			
≤II	6 (27%)	5 (19%)	0.732
≥III	16 (73%)	21 (81%)	
Medical therapy	Loop diuretics – 6 (27%) MRA – 5 (23%) PDE5i – 20 (91%) ETRA – 16 (73%) Riociguat – 2 (9%) Selexipag – 1 (5%) Inhaled Illoprost – 2 (9%) IV epoprostenol – 3 (14%) DOAC – 2 (9%) Warfarin – 3 (14%)	Loop diuretics – 19 (73%) Thiazide diuretics – 2 (8%) MRA – 7 (27%) PDE5i – 3 (12%) Beta-blocker – 17 (65%) ACEi/ARB – 9 (35%) CCB – 6 (23%) Digoxin – 5 (19%) Statin – 13 (50%) DOAC – 9 (35%) Warfarin – 10 (38%)	**–**
Catheterization-CMR interval (days)	3 (1–63)	1 (0–7)	0.154
Catheterization-TTE interval (days)	33 (1–110)	36 (22–64)	0.626
TTE-CMR interval (days)	18 (1–43)	39 (22–78)	**0.025**

*PAH, pulmonary arterial hypertension; CTD, connective tissue disease; HIV, human immunodeficiency virus; CHD, congenital heart disease; SD, systolic dysfunction (LVEF < 50%); DD, diastolic dysfunction; MRA, mineralocorticoid receptor antagonist; ETRA, endothelin receptor antagonist (ambrisentan and macitentan); DOAC, direct oral anticoagulant; ACEi/ARB, angiotensin-converting enzyme inhibitor/angiotensin receptor blocker; CCB, calcium channel blocker; PDE5i, phosphodiesterase type 5 inhibitor (sildenafil and tadalafil). The bold values indicate a p-value of ≤ 0.05*.

### Hemodynamic Findings

Mean PA pressures (mPAP) were higher in the pre-capillary cohort as was PVR. Cardiac index (CI) was similar between groups. Mean RA pressures (mRAP) were higher in post-capillary PH patients (Table 2).

**Table 2:** Invasive haemodynamic parameters.

	**Pre-capillary** **(*n* = 22)**	**Post-capillary** **(*n* = 26)**	***p*-value**
PA systolic (mmHg)	89 ± 21	65 ± 17	**<0.001**
PA diastolic (mmHg)	36 ± 13	26 ± 6	**0.002**
mPAP (mmHg)	55 ± 14	42 ± 9	**<0.001**
mPAWP (mmHg)	10 ± 3	22 ± 5	**<0.001**
mRAP (mmHg)	9 ± 6	14 ± 5	**0.003**
PVR (Wood units)	11.7 (7.8–18.0)	3.7 (2.4–5.7)	**<0.001**
SaO_2_ (%)	95.9 ± 2.7	95.3 ± 3.0	0.450
SvO_2_ (%)	68.0 ± 6.6	69.8 ± 8.7	0.419
Stroke volume indexed (ml/m^2^)	29 (25–33)	31 (28–38)	0.106
Cardiac index (L/min/m^2^)	2.0 (1.8–2.5)	2.1 (1.9–2.6)	0.282
HR (bpm)	80 ± 15	75 ± 11	0.227

*The bold values indicate a p-value of ≤ 0.05*.

### Imaging Findings

#### Echocardiography

TR Vmax was lower but transmitral E wave velocity was higher in post-capillary PH patients. ePLAR and all ePLAR variants were significantly higher in pre-capillary patients in context of greater TR Vmax and lower LV filling pressure estimates by E/e'. Lastly, TTE LAVi was smaller in the pre-capillary group (Table 3).

**Table 3:** TTE parameters.

	**Pre-capillary** **(*n* = 22)**	**Post-capillary** **(*n* = 26)**	***p*-value**
TR Vmax (m/s)	4.2 ± 0.8	3.7 ± 0.5	**0.009**
Transmitral E (cm/s)	56 ± 19	96 ± 28	**<0.001**
Medial e' (cm/s)	6 ± 2	6 ± 2	0.785
Lateral e' (cm/s)	11 ± 4	9 ± 3	0.223
E/medial e'	10.0 ± 3.9	17.9 ± 8.7	**<0.001**
E/lateral e'	5.8 ± 2.7	11.1 ± 4.7	**<0.001**
Average E/e'	7.9 ± 2.9	14.5 ± 6.3	**<0.001**
ePLAR (E/medial e')	0.48 (0.29–0.59)	0.22 (0.17–0.34)	**<0.001**
Variant ePLAR (E/lateral e')	0.79 (0.51–1.10)	0.36 (0.27–0.47)	**<0.001**
Variant ePLAR (average E/e')	0.51 (0.39–0.76)	0.25 (0.21–0.37)	**<0.001**
LAVi (ml/m^2^)	23 ± 7	45 ± 17	**<0.001**

*The bold values indicate a p-value of ≤ 0.05*.

#### CMR

Pre-capillary PH patients had smaller left atria and ventricles relative to the right. The post-capillary group had significantly larger left atria. There was no difference in RAVi between groups, however peak RA strain was lower in post-capillary patients (Table 4).

**Table 4:** CMR volumetric and FT strain assessment.

	**Pre-capillary** **(*n* = 22)**	**Post-capillary** **(*n* = 26)**	***p*-value**
LAVi (ml/m^2^)	25 ± 9	60 ± 22	**<0.001**
Peak LA strain (%)	22.6 ± 8.4	9.0 ± 5.8	**<0.001**
RAVi (ml/m^2^)	60 ± 21	61 ± 29	0.694
Peak RA strain (%)	18.8 ± 7.0	8.9 ± 5.5	**<0.001**
LA/RA volume ratio	0.33 (0.29–0.65)	1.0 (0.83–1.30)	**<0.001**
LVEDVi (ml/m^2^)	48 (43–54)	64 (46–73)	**0.018**
LVESVi (ml/m^2^)	17 (16–25)	22 (15–30)	0.178
LV stroke volume indexed (ml/m^2^)	30 (26–34)	36 (29–43)	**0.006**
LVEF (%)	62 (59–66)	63 (59–67)	0.656
LV GLS (%)	−13.3 ± 3.2	−13.4 ± 2.9	0.910
RVEDVi (ml/m^2^)	119 (97–136)	76 (69–88)	**<0.001**
RVESVi (ml/m^2^)	82 (61–98)	39 (31–50)	**<0.001**
RV stroke volume indexed (ml/m^2^)	39 (34–44)	38 (30–50)	0.885
RVEF (%)	35 ± 9	49 ± 9	**<0.001**
RVFWLS (%)	−10.1 ± 3.7	−13.7 ± 3.1	**<0.001**
LVEDV/RVEDV ratio	0.44 (0.32–0.56)	0.78 (0.62–0.92)	**<0.001**

*The bold values indicate a p-value of ≤ 0.05*.

Amongst post-capillary patients, there was commensurately severe biatrial impairment of peak strain (LA 9.0 ± 5.8%, RA 8.9 ± 5.5%).

All patients had preserved LVEF however longitudinal function assessed by LV GLS was similarly impaired in both groups. RVEF was more impaired in pre-capillary patients with concomitant/proportional RVFWLS reduction.

### Distinguishing Post- From Pre-capillary PH

Ten parameters (4 TTE, 6 CMR) were assessed for their ability to distinguish post- from pre-capillary PH.

**Figure 2:**
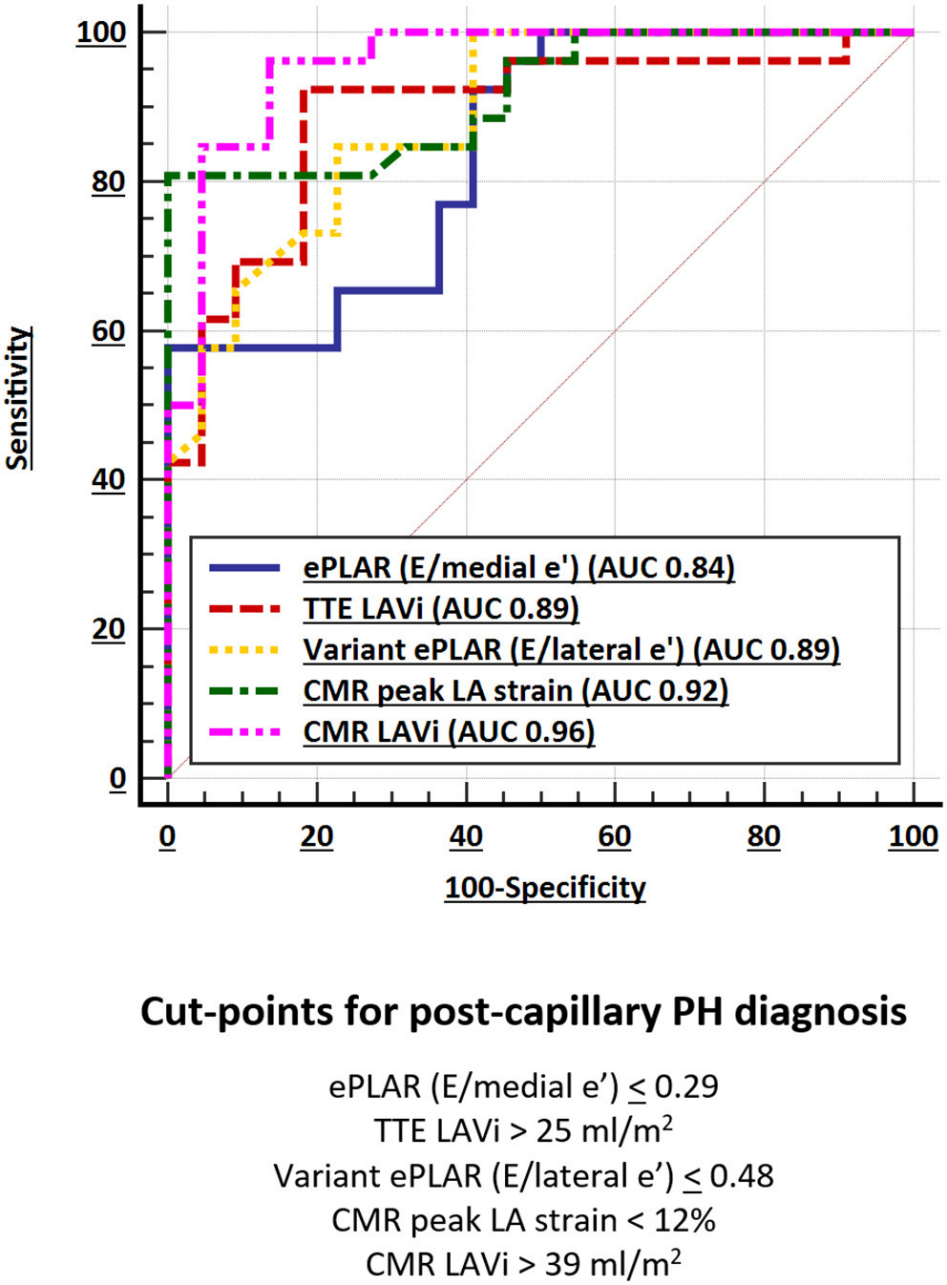
Comparison of selected TTE and CMR discriminator AUC.

Of all (TTE and CMR) parameters, CMR LAVi had the greatest AUC (0.96) followed by peak LA strain (AUC 0.92) (Figure 2; Table 5).

**Table 5:** Comparison of predictive ability/AUC of 10 discriminator parameters with associated cut-points for post-capillary PH diagnosis.

	**AUC (95% CI)^†^**	**Cut-point for** **post-capillary PH**	**+LR**	**–LR**	**Sens**	**Spec**
**TTE**						
ePLAR (E/medial e')	0.84 (0.71–0.93)	≤0.29	2.9	0.5	65%	77%
Variant ePLAR (E/lateral e')	0.89 (0.76–0.96)	≤0.48	3.7	0.2	85%	77%
Variant ePLAR (average E/e')	0.88 (0.75–0.95)	≤0.39	4.4	0.2	81%	82%
TTE LAVi (ml/m^2^)	0.89 (0.77–0.96)	>25	5.1	0.1	92%	82%
**CMR**						
Peak LA strain (%)	0.92 (0.80–0.98)	<12	–	0.2	81%	100%
CMR LAVi (ml/m^2^)	0.96 (0.86–1.00)	>39	18.6	0.2	85%	95%
LA/RA volume ratio	0.91 (0.79–0.97)	>0.72	4.9	0.1	88%	82%
LVEDV/RVEDV ratio	0.87 (0.75–0.95)	>0.56	4.4	0.2	81%	82%
RVFWLS (%)	0.87 (0.74–0.95)	≤-10.4	2.2	0.2	88%	59%
RVEF (%)	0.76 (0.62–0.87)	>42	4.2	0.3	77%	82%

† *All p < 0.001*.

Echocardiographic parameters also had excellent, albeit smaller AUC. ePLAR (E/medial e') had the smallest AUC of all (0.84) while variant ePLAR (E/lateral e') and TTE LAVi performed similarly well (AUC 0.89).

Consistent with prior data (6), a CMR LAVi of >39 ml/m^2^ had 85% sensitivity and 95% specificity for distinguishing post- from pre-capillary PH. Compared to CMR LAVi, a peak LA strain cut-point of <12% had excellent sensitivity at 81% and greater specificity at 100% (Table 5). Stepwise logistic regression showed that CMR LAVi and peak LA strain had a combined multivariate AUC of 0.98 (95% CI 0.89–1.00; *p* < 0.001) for post-capillary PH diagnosis.

### Comparison of TTE and CMR LAV

**Figure 3:**
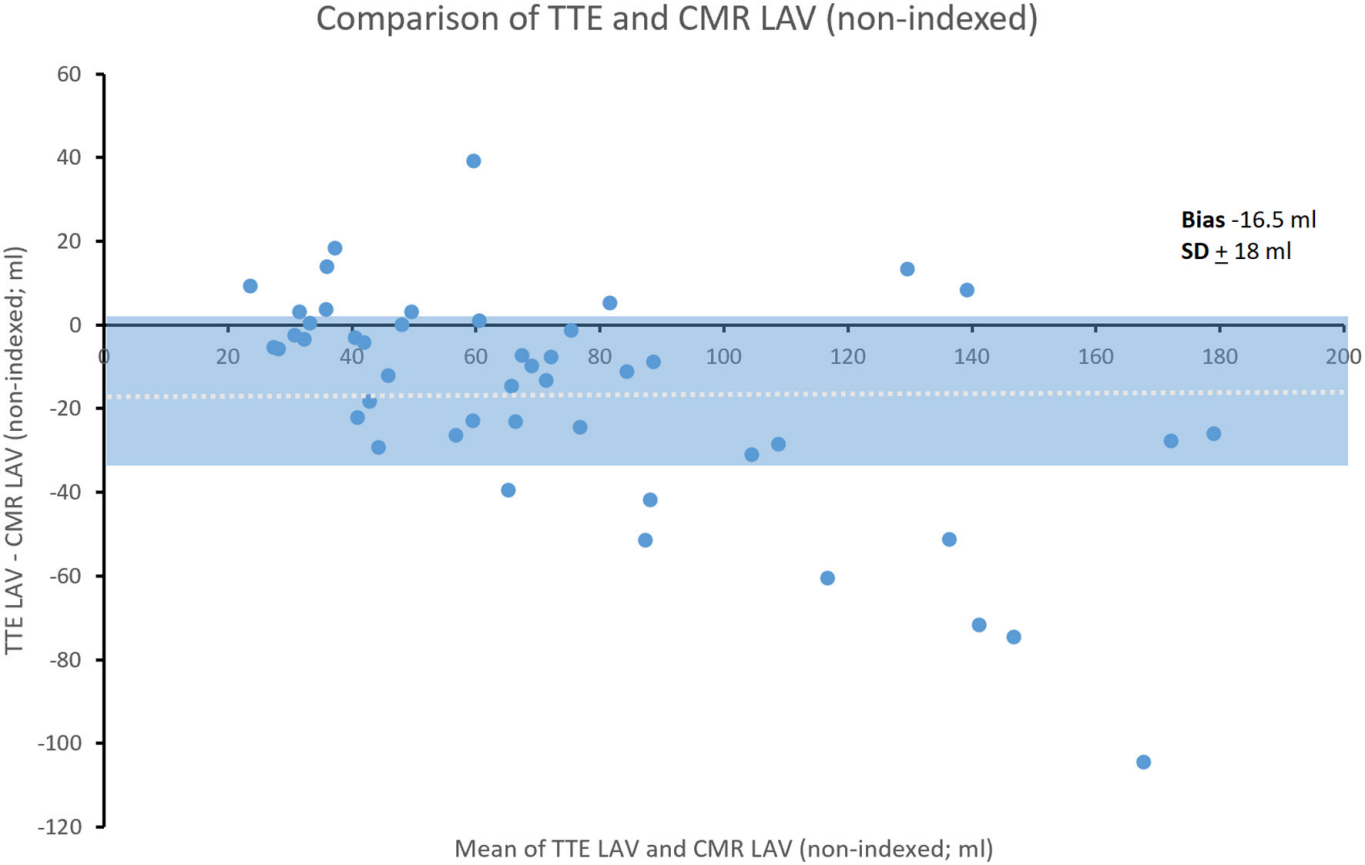
Bland-Altman comparison of TTE and CMR non-indexed LAV. Shading represents ± SD. Dotted line indicates mean bias.

Non-indexed LAV was underestimated by TTE compared to CMR (bias ± SD; −16.5 ± 18 ml) (Figure 3).

### Correlation Analysis

**Figure 4:**
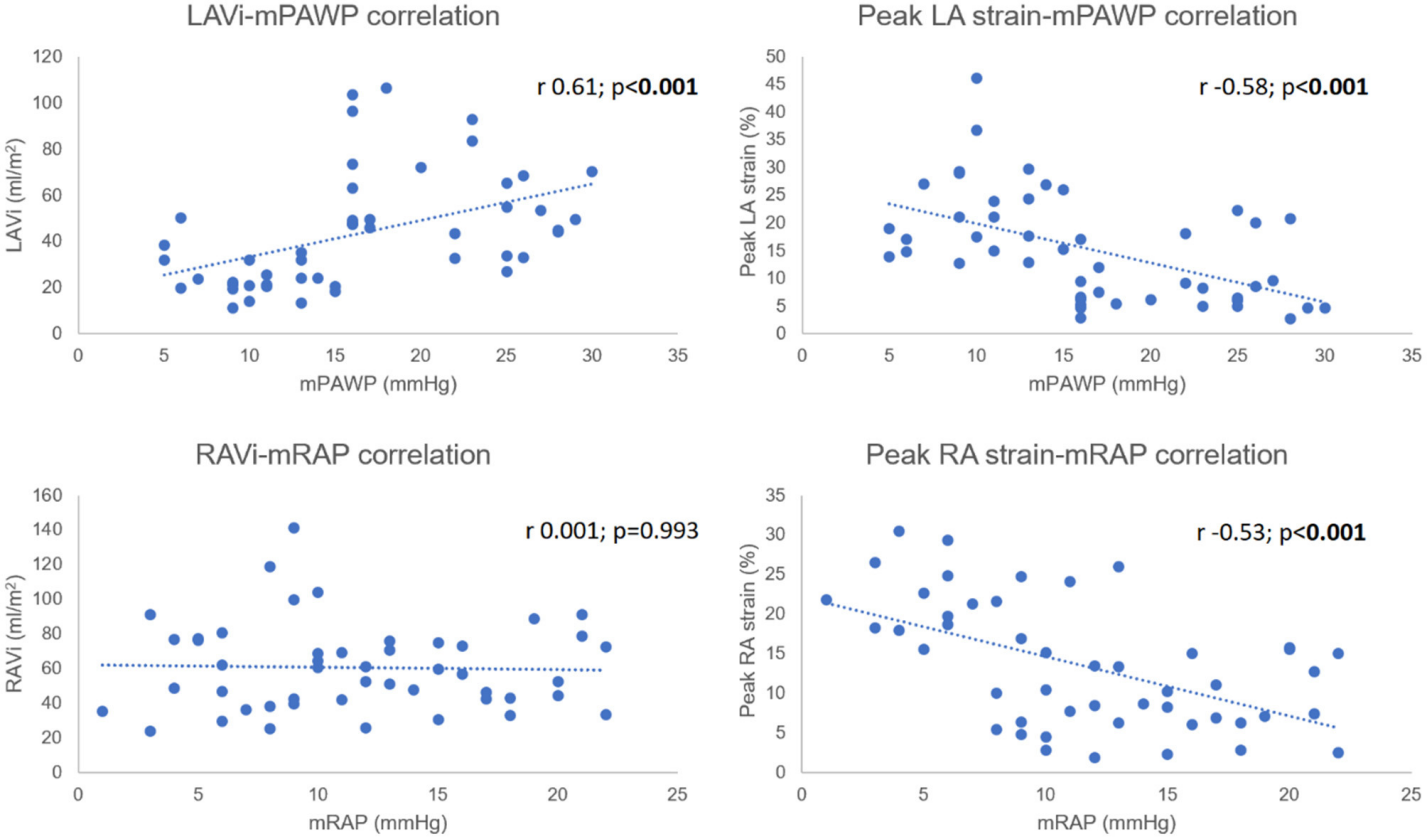
Biatrial pressure-volumetric/strain correlation in entire cohort.

Both CMR LAVi (r 0.61, 95% CI 0.39–0.76) and peak LA strain (r −0.58, 95% CI −0.75 to −0.36) were significantly correlated with mPAWP (both *p* < 0.001). A similar relationship was seen for mRAP and peak RA strain (r −0.53, 95% CI −0.71 to −0.30; *p* < 0.001). There was however no association between mRAP and RAVi (r 0.001, 95% CI −0.28 to 0.29; *p* = 0.993) (Figure 4).

**Figure 5:**
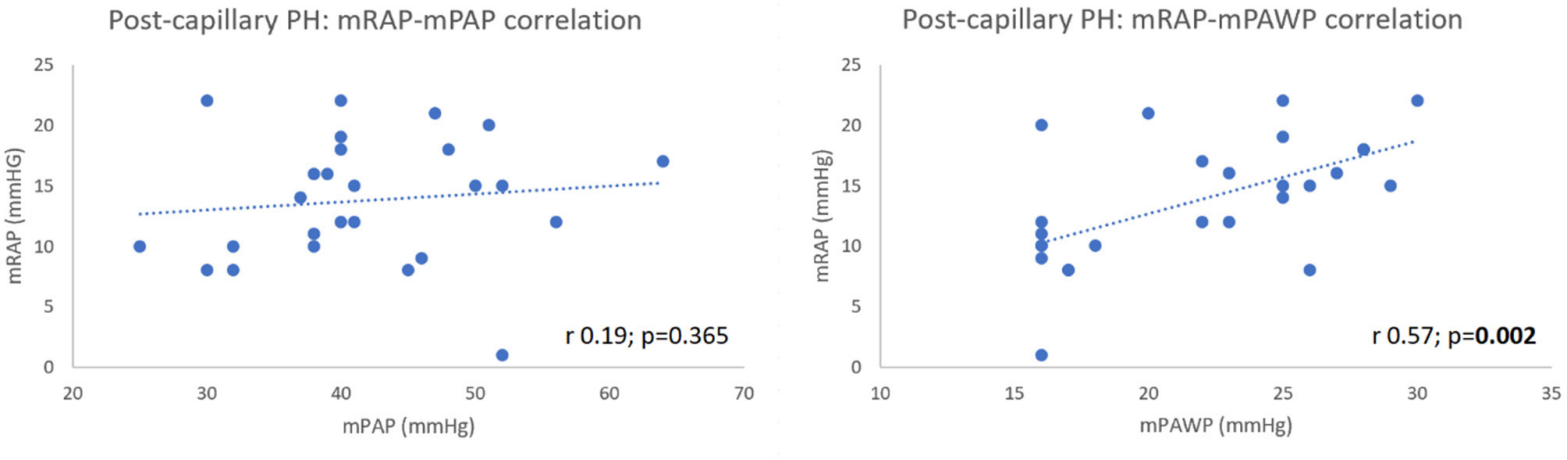
mRAP-mPAP and mRAP-mPAWP relationships in post-capillary PH patients.

Amongst post-capillary PH patients, there was a significant positive correlation between mRAP and mPAWP (r 0.57, 95% CI 0.24–0.79; *p* = 0.002) (Figure 5) which was stronger than that observed in the pre-capillary group (r 0.50, 95% CI 0.09–0.76; *p* = 0.019). Lastly, there was no correlation between mRAP and mPAP in post-capillary patients (r 0.19, 95% CI −0.22 to 0.53; *p* = 0.365) (Figure 5).

## Discussion

In this work, we have shown that CMR LA volumetric and deformation assessment can accurately distinguish post- from pre-capillary PH and was superior to TTE. LA volumetric assessment was more sensitive but deformation assessment more specific and diagnostic accuracy was enhanced with use of both parameters (multivariate AUC 0.98).

TTE's predictive performance for post-capillary PH was optimized when variant ePLAR (E/lateral e') was utilized, with an augmented AUC of 0.89 compared to ePLAR (E/medial e'; AUC 0.84).

The discriminatory ability of LAV was maximized as a CMR parameter, circumventing TTE limitations of suboptimal acoustic windows with certain body habitus. Additionally, the LA long axis plane is typically divergent from that of the LV, causing TTE volumetric underestimation without atrial-focussed imaging (21, 22).

This is also the first study to contrast biatrial and biventricular CMR FT deformation parameters in the same PH cohort. There are several notable findings.

In a canine PH model by PA banding, early compensation was characterized by preserved systolic but impaired RV diastolic function, and increased RA distensibility (increased reservoir strain) (23). The latter would be counterpoise for RV diastolic dysfunction and avert clinical heart failure but with worsening PH severity/chronicity, RV-PA then RV-RA uncoupling would ensue with diminution of RA reservoir function causing symptoms (24, 25). Strain cut-points have been proposed to accompany this trajectory of maladaptation (24, 26, 27) with RV-PA decoupling occurring at RVFWLS > −15.29% and later stage RV-RA decoupling corresponding to a RA reservoir strain < 16%. In this cross-sectional work, pre-capillary PH patients had a mean RVFWLS of −10.1% and peak RA strain of 18.8% suggesting uncoupled RV-PA but preserved RV-RA relationships.

Both pre- and post-capillary PH patients had similarly severe RA dilatation however post-capillary patients had lower RA peak strain and higher mRAP despite the similar volumes.

Tello et al. (24) have shown peak RA strain to reflect RV diastolic rather than systolic function and our data would imply more pronounced RV diastolic dysfunction in post-capillary PH patients compared to the pre-capillary group.

Right ventricular diastolic dysfunction in left heart disease is poorly characterized. Independent of RVSP elevation, Yu et al. (28) have demonstrated similar magnitude diastolic abnormalities of trans-tricuspid inflow in HFrEF patients without PH (normal RVSP) and PH patients (LVEF > 55%; mainly COPD and valvular heart disease). This would suggest a separate/additional mechanism for RV diastolic dysfunction other than PH. Importantly, a close correlation existed between RV and LV diastolic parameters but not between RV diastolic function and LV size/systolic function.

Recent data from Patel et al. (29) indicate that whilst CMR RV extracellular volume (ECV) increment was seen to a similar degree in PAH and post-capillary PH patients, a significant correlation between RV ECV and PVR was only seen in PAH patients. This would again suggest additional PH-independent mechanisms contributing to diffuse RV fibrosis in post-capillary patients.

Su et al. (30) has shown significant correlation between LV diastolic dysfunction and diffuse LV myocardial fibrosis (by CMR ECV) in HFpEF. In a porcine CTEPH model (31), RV afterload elevation was associated with increased RV expression of pro-fibrotic genes. Notably, the latter was not observed in the LV, with no change in LV myocardial interstitial collagen content.

Summarily, it would appear that biventricular fibrosis is seen in post-capillary PH whereas RV fibrosis predominates in pre-capillary PH.

Our work adds further to these data. Our post-capillary cohort comprised mainly of HFpEF patients with commensurately severe biatrial peak strain reduction. As depicted in Figure 5, mRAP was significantly correlated with mPAWP but not with mPAP in post-capillary PH patients. Post-capillary PH patients had greater reduction in RA strain than pre-capillary patients despite the latter having higher mPAP.

These observations would suggest that clinically significant RV diastolic dysfunction occurs in both pre- and post-capillary PH however in pre-capillary patients, when symptomatic, represents late stage disease (RV-RA uncoupling) following RV-PA uncoupling in the context of progressive afterload elevation (24). Conversely in post-capillary PH, we propose that RV diastolic dysfunction is an earlier event, likely coincident with and reflective of LV diastolic dysfunction in keeping with the paradigm of ventricular interdependence (32) rather than simply reflecting transmitted pressures across the pulmonary circulation.

In proposing additional mechanistic hypotheses for the dissimilarity in atrial strain between both groups, it is also important to note that the majority of post-capillary patients had a history of AF. Longstanding AF leads to atrial structural change/diffuse fibrosis causing impairment of atrial phasic function and peak reservoir strain, with the magnitude of strain impairment corresponding to the degree of atrial fibrosis (33, 34).

Beyond AF itself (“AF begets AF”), post-capillary PH patients also had multiple contributors of atrial arrhythmogenic remodeling (35) including demography (older age; post-capillary mean age 71 years vs. pre-capillary mean age 48 years), higher body weight and co-morbidities (e.g., post-capillary mean serum creatinine 114 μmol/L vs. pre-capillary mean 77 μmol/L). These factors would further diminish atrial function and underpin our observations.

Both groups had similarly preserved LVEF but reduced GLS. The pathophysiology underlying this subclinical dysfunction is likely disparate between groups. In pre-capillary PH patients with higher mPAP, PVR, RV volumes and greater RV dysfunction, abnormal septal configuration and reduced LV preload are likely principal mechanisms for LV GLS impairment (36, 37). In post-capillary PH patients, commonly older and more co-morbid, the dominant mechanism is likely diffuse myocardial fibrosis (30, 38–40).

Lastly, our data contributes to the literature regarding determination of congruence between CMR and TTE deformation assessment. Inoue and colleagues (41) showed similar findings of peak LA strain reduction in post-capillary (vs. pre-capillary) PH patients using speckle tracking TTE, and proposed a comparable discriminatory peak strain cut-point. Our utilization of ubiquitous commercial software for CMR FT strain assessment, instead of proprietary in-house methods, would also improve the “real-world” applicability of our findings.

Subsequent to this, prospective validation of the diagnostic performance of combined CMR LA volumetric and strain assessment may enhance the current PH workup algorithm. Study into the prognostic value of CMR FT strain in PH would also be invaluable in view of the present paucity of imaging prognostic markers, specifically with examination of change in deformation parameters following targeted therapy.

## Conclusion

Comprehensive CMR FT deformation assessment offers additional physiologic insights in PH beyond sole volumetric evaluation and may enhance the current PH workup algorithm.

## Limitations

This was a retrospective study from a single institution. The relatively small cohort size (*n* = 48) was due to exclusion of a large proportion of patients who had part of their PH workup completed externally prior to referral. Age and gender matching of pre- and post-capillary groups was not possible due to the typical disparate demography of PAH and diastolic dysfunction/HFpEF patients. There was a significant interval between all investigations, with potential for change in loading conditions with medical therapy however this does not appear to have affected the predictive ability of assessed TTE and CMR parameters. The strength of imaging-hemodynamic correlation was also maintained and is consistent with published data. Additionally, the time interval reflects current actual service provision at our institution. Amongst post-capillary PH patients, there was a larger proportion with an AF history but this would be congruent with the post-capillary phenotype. With CMR FT LA strain, we acknowledge the limitation of single cardiac cycle atrial strain assessment in AF compared to averaging in TTE but again, this does not appear to have affected the predictive ability of CMR LA strain. Lastly, atrial strain was not assessed using a dedicated algorithm but rather by a ventricular algorithm from a specific vendor.

## Data Availability Statement

The raw data supporting the conclusions of this article will be made available by the authors, without undue reservation.

## Ethics Statement

The studies involving human participants were reviewed and approved by Health Research Authority and NHS Research Ethics Service. Written informed consent for participation was not required for this study in accordance with the national legislation and the institutional requirements.

## Author Contributions

KL, LH, DG, and SG contributed to conception and design of the study. KL organized the database and wrote the first draft of the manuscript. KL and DG performed the image analysis. HP and KL performed the statistical analysis. LH, FL, GH, and RD wrote sections of the manuscript. All authors contributed to manuscript revision, read, and approved the submitted version.

## Conflict of Interest

The authors declare that the research was conducted in the absence of any commercial or financial relationships that could be construed as a potential conflict of interest.

## Publisher's Note

All claims expressed in this article are solely those of the authors and do not necessarily represent those of their affiliated organizations, or those of the publisher, the editors and the reviewers. Any product that may be evaluated in this article, or claim that may be made by its manufacturer, is not guaranteed or endorsed by the publisher.

## References

[1] Galie N Humbert M Vachiery JL Gibbs S Lang I Torbicki A. 2015 ESC/ERS guidelines for the diagnosis and treatment of pulmonary hypertension: the joint task force for the diagnosis and treatment of pulmonary hypertension of the European Society of Cardiology (ESC) and the European Respiratory Society (ERS): Endorsed by: Association for European Paediatric and Congenital Cardiology (AEPC), International Society for Heart and Lung Transplantation (ISHLT). Eur Respir J. (2015) 46:903–75. 10.1183/13993003.01032-2015 26318161

[2] Simonneau G Montani D Celermajer DS Denton CP Gatzoulis MA Krowka M. Haemodynamic definitions and updated clinical classification of pulmonary hypertension. Eur Respir J. (2019) 53:1801913. 10.1183/13993003.01913-2018 30545968 PMC6351336

[3] Farber HW Gibbs S. Under pressure: pulmonary hypertension associated with left heart disease. Eur Respir Rev. (2015) 24:665–73. 10.1183/16000617.0059-2015 26621980 PMC9487627

[4] Scalia GM Scalia IG Kierle R Beaumont R Cross DB Feenstra J. ePLAR - the echocardiographic pulmonary to left atrial ratio - a novel non-invasive parameter to differentiate pre-capillary and post-capillary pulmonary hypertension. Int J Cardiol. (2016) 212:379–86. 10.1016/j.ijcard.2016.03.035 27061467

[5] Venkateshvaran A Manouras A Kjellstrom B Lund HL. The additive value of echocardiographic pulmonary to left atrial global strain ratio in the diagnosis of pulmonary hypertension. Int J Cardiol. (2019) 292:205–10. 10.1016/j.ijcard.2019.05.025 31176524

[6] Crawley SF Johnson MK Dargie HJ Peacock JA. LA volume by CMR distinguishes idiopathic from pulmonary hypertension due to HFpEF. JACC Cardiovasc Imaging. (2013) 6:1120–1. 10.1016/j.jcmg.2013.05.014 24135327

[7] Huis In 't Veld AE Van Vliet AG Spruijt OA Handoko ML Marcus JT Vonk Noordegraaf A. CTA-derived left to right atrial size ratio distinguishes between pulmonary hypertension due to heart failure and idiopathic pulmonary arterial hypertension. Int J Cardiol. (2016) 223:723–8. 10.1016/j.ijcard.2016.08.314 27573596

[8] Ayach B Fine NM Rudski GL. Right ventricular strain: measurement and clinical application. Curr Opin Cardiol. (2018) 33:486–92. 10.1097/HCO.0000000000000540 30063529

[9] Potter E Marwick TH. Assessment of left ventricular function by echocardiography: the case for routinely adding global longitudinal strain to ejection fraction. JACC Cardiovasc Imaging. (2018) 11:260–74. 10.1016/j.jcmg.2017.11.017 29413646

[10] Vo HQ Marwick TH Negishi K. MRI-derived myocardial strain measures in normal subjects. JACC Cardiovasc Imaging. (2018) 11:196–205. 10.1016/j.jcmg.2016.12.025 28528164

[11] Lundberg A Johnson J Hage C Back M Merkely B Venkateshvaran A. Left atrial strain improves estimation of filling pressures in heart failure: a simultaneous echocardiographic and invasive haemodynamic study. Clin Res Cardiol. (2019) 108:703–15. 10.1007/s00392-018-1399-8 30536044 PMC6529379

[12] Cameli M Sparla S Losito M Righini FM Menci D Lisi M. Correlation of left atrial strain and Doppler measurements with invasive measurement of left ventricular end-diastolic pressure in patients stratified for different values of ejection fraction. Echocardiography. (2016) 33:398–405. 10.1111/echo.13094 26493278

[13] Singh A Medvedofsky D Mediratta A Balaney B Kruse E Ciszek B. Peak left atrial strain as a single measure for the non-invasive assessment of left ventricular filling pressures. Int J Cardiovasc Imaging. (2019) 35:23–32. 10.1007/s10554-018-1425-y 30062535 PMC6353699

[14] Lang RM Badano LP Mor-Avi V Afilalo J Armstrong A Ernande L. Recommendations for cardiac chamber quantification by echocardiography in adults: an update from the American Society of Echocardiography and the European Association of Cardiovascular Imaging. J Am Soc Echocardiogr. (2015) 28:1–39.e14. 10.1016/j.echo.2014.10.003 25559473

[15] Ruan Q Nagueh SF. Clinical application of tissue Doppler imaging in patients with idiopathic pulmonary hypertension. Chest. (2007) 131:395–401. 10.1378/chest.06-1556 17296639

[16] Nagueh SF Smiseth OA Appleton CP Byrd BF Dokainish H Edvardsen T. Recommendations for the evaluation of left ventricular diastolic function by echocardiography: an update from the American Society of Echocardiography and the European Association of Cardiovascular Imaging. Eur Heart J Cardiovasc Imaging. (2016) 17:1321–60. 10.1093/ehjci/jew082 27422899

[17] Schulz-Menger J Bluemke DA Bremerich J Flamm SD Fogel MA Friedrich MG. Standardized image interpretation and post-processing in cardiovascular magnetic resonance - 2020 update : Society for Cardiovascular Magnetic Resonance (SCMR): Board of Trustees Task Force on Standardized Post-Processing. J Cardiovasc Magn Reson. (2020) 22:19. 10.1186/s12968-020-00610-6 32160925 PMC7066763

[18] Truong VT Palmer C Wolking S Sheets B Young M Ngo TNM. Normal left atrial strain and strain rate using cardiac magnetic resonance feature tracking in healthy volunteers. Eur Heart J Cardiovasc Imaging. (2020) 21:446–453. 10.1093/ehjci/jez157 31504357

[19] Truong VT Palmer C Young M Wolking S Ngo TNM Sheets B. Right Atrial Deformation Using Cardiovascular Magnetic Resonance Myocardial Feature Tracking Compared with Two-Dimensional Speckle Tracking Echocardiography in Healthy Volunteers. Sci Rep. (2020) 10:5237. 10.1038/s41598-020-62105-9 32251322 PMC7089993

[20] Bistoquet A Oshinski J Skrinjar O. Myocardial deformation recovery from cine MRI using a nearly incompressible biventricular model. Med Image Anal. (2008) 12:69–85. 10.1016/j.media.2007.10.009 18234539

[21] Addetia K Lang RM. Complexities of left atrial analysis: more than meets the eye? Circ Cardiovasc Imaging. (2016) 9:e005196. 10.1161/CIRCIMAGING.116.005196 27412661

[22] Badano LP Miglioranza MH Mihaila S Peluso D Xhaxho J Marra MP. Left atrial volumes and function by three-dimensional echocardiography: reference values, accuracy, reproducibility, and comparison with two-dimensional echocardiographic measurements. Circ Cardiovasc Imaging. (2016) 9:e004229. 10.1161/CIRCIMAGING.115.004229 27412658

[23] Gaynor SL Maniar HS Bloch JB Steendijk P Moon RM. Right atrial and ventricular adaptation to chronic right ventricular pressure overload. Circulation. (2005) 112 (9 Suppl.):I212–8. 10.1161/CIRCULATIONAHA.104.517789 16159819

[24] Tello K Dalmer A Vanderpool R Ghofrani HA Naeije R Roller F. Right ventricular function correlates of right atrial strain in pulmonary hypertension: a combined cardiac magnetic resonance and conductance catheter study. Am J Physiol Heart Circ Physiol. (2020) 318:H156–64. 10.1152/ajpheart.00485.2019 31756118

[25] Lang IM Binder T. Right atrial strain is a surrogate of coupling in the right heart. Eur Heart J Cardiovasc Imaging. (2020) 21:863–4. 10.1093/ehjci/jeaa104 32412596

[26] Vonk Noordegraaf A Chin KM Haddad F Hassoun PM Hemnes AR Hopkins SR. Pathophysiology of the right ventricle and of the pulmonary circulation in pulmonary hypertension: an update. Eur Respir J. (2019) 53:1801900. 10.1183/13993003.01900-2018 30545976 PMC6351344

[27] Tello K Dalmer A Vanderpool R Ghofrani HA Naeije R Roller F. Cardiac magnetic resonance imaging-based right ventricular strain analysis for assessment of coupling and diastolic function in pulmonary hypertension. JACC Cardiovasc Imaging. (2019) 12:2155–2164. 10.1016/j.jcmg.2018.12.032 30878422

[28] Yu CM Sanderson JE Chan S Yeung L Hung YT Woo SK. Right ventricular diastolic dysfunction in heart failure. Circulation. (1996) 93:1509–14. 10.1161/01.CIR.93.8.1509 8608618

[29] Patel RB Li E Benefield BC Swat SA Polsinelli VB Carr JC. Diffuse right ventricular fibrosis in heart failure with preserved ejection fraction and pulmonary hypertension. ESC Heart Fail. (2020) 7:253–63. 10.1002/ehf2.12565 31903694 PMC7083501

[30] Su MY Lin LY Tseng YH Chang CC Wu CK Lin JL. CMR-verified diffuse myocardial fibrosis is associated with diastolic dysfunction in HFpEF. JACC Cardiovasc Imaging. (2014) 7:991–7. 10.1016/j.jcmg.2014.04.022 25240451

[31] Stam K Cai Z van der Velde N van Duin R Lam E van der Velden J. Cardiac remodelling in a swine model of chronic thromboembolic pulmonary hypertension: comparison of right vs. left ventricle. J Physiol. (2019) 597:4465–80. 10.1113/JP277896 31194256 PMC6852085

[32] Clyne CA Alpert JS Benotti RJ. Interdependence of the left and right ventricles in health and disease. Am Heart J. (1989) 117:1366–73. 10.1016/0002-8703(89)90418-3 2658522

[33] Tops LF Delgado V Bertini M Marsan NA Den Uijl DW Trines SA. Left atrial strain predicts reverse remodeling after catheter ablation for atrial fibrillation. J Am Coll Cardiol. (2011) 57:324–31. 10.1016/j.jacc.2010.05.063 21232671

[34] Kuppahally SS Akoum N Badger TJ Burgon NS Haslam T Kholmovski E. Echocardiographic left atrial reverse remodeling after catheter ablation of atrial fibrillation is predicted by preablation delayed enhancement of left atrium by magnetic resonance imaging. Am Heart J. (2010) 160:877–84. 10.1016/j.ahj.2010.07.003 21095275 PMC2995281

[35] Nattel S Harada M. Atrial remodeling and atrial fibrillation: recent advances and translational perspectives. J Am Coll Cardiol. (2014) 63:2335–45. 10.1016/j.jacc.2014.02.555 24613319

[36] Kallianos K Brooks GC Mukai K Seguro de Carvalho F Liu J Naeger DM. Cardiac magnetic resonance evaluation of left ventricular myocardial strain in pulmonary hypertension. Acad Radiol. (2018) 25:129–35. 10.1016/j.acra.2017.07.009 28866441 PMC5723230

[37] Gurudevan SV Malouf PJ Auger WR Waltman TJ Madani M Raisinghani AB. Abnormal left ventricular diastolic filling in chronic thromboembolic pulmonary hypertension: true diastolic dysfunction or left ventricular underfilling? J Am Coll Cardiol. (2007) 49:1334–9. 10.1016/j.jacc.2007.01.028 17394966

[38] DeVore AD McNulty S Alenezi F Ersboll M Vader JM Oh JK. Impaired left ventricular global longitudinal strain in patients with heart failure with preserved ejection fraction: insights from the RELAX trial. Eur J Heart Fail. (2017) 19:893–900. 10.1002/ejhf.754 28194841 PMC5511088

[39] Kosmala W Przewlocka-Kosmala M Szczepanik-Osadnik H Mysiak A Marwick HT. Fibrosis and cardiac function in obesity: a randomised controlled trial of aldosterone blockade. Heart. (2013) 99:320–6. 10.1136/heartjnl-2012-303329 23343682

[40] Mochizuki Y Tanaka H Matsumoto K Sano H Toki H Shimoura H. Clinical features of subclinical left ventricular systolic dysfunction in patients with diabetes mellitus. Cardiovasc Diabetol. (2015) 14:37. 10.1186/s12933-015-0201-8 25889250 PMC4404084

[41] Inoue K Remme EW Khan FH Andersen OS Gude E Skulstad H. P2452Application of left atrial strain for differentiation between pre- and post-capillary pulmonary hypertension. Eur Heart J. (2019) 40 (Suppl.1). 10.1093/eurheartj/ehz748.0784

